# Report on Pathology and Microscopy

**Published:** 1874-06

**Authors:** I. N. Danforth

**Affiliations:** Lecturer on Pathology and Microscopy, Rush Medical College


ARTICLE I.—*Report on Pathology and Microscopy.* By I. N. DANFORTH, M.D., Lecturer on Pathology and Microscopy, Rush Medical College.

1. Spindle-Celled Sarcoma. Dr. B. JOY JEFFRIES. Prof. LANKFORD.

2. Bright's Disease, with Leucocythæmia and Disease of Bone Marrow. By Dr. H. C. WOOD, Philadelphia Hospital.

3. A Singular Case of Gout. (*Medical Record*, Jan. 1.)

4. The Origin and Action of Miasm. (*New Orleans Medical and Surgical Journal*, Jan., 1874.

5. Bacteria in the Blood. Dr. EBERTH. (*St. Louis Med. and Surg. Jour.*, March, 1874.)

6. Ascites from Obstruction in the Portal Vein. Sir W. JENNER. (*Practitioner*, London, Feb., 1874.)

7. Embolism of the Arteries of the Extremities. Dr. SAMUEL B. WARD. (*New York Med. Jour.*, March, 1874.)

8. Embolism of Pulmonary Artery. Dr. LENTE. (*The Medical and Surgical Reporter*, March 14, 1874.)

1. Dr. B. Joy Jeffries, of Boston, describes an intra-ocular spindle-celled sarcoma, removed by himself on the 1st of April last, in the following language:

"The substance of the tumor was firm, homogeneous, whitish, and no pigment was seen. It was attached to the sclerotic, about the size of a small filbert, reaching forward to the insertion of the iris, and backward beyond the median line. It touched the back of the lens, pressing forward the ciliary muscles and processes. The sclerotic lying against it was perfect. It occupied the place of the choroid, pushing the retina inward on its surface. The rest of the globe and optic nerve seemed perfectly normal. The microscope showed it to be a fusiformed-cell white sarcoma, such as Knapp gives in Tab. XII, Figs. 50–53, in his monograph on intra-ocular tumors." (Trans. Am. Ophth. Soc., 1873.)

Prof. Lankford, of the Missouri Medical College, amputated the thigh of a man aged seventy-four recently, on account of a spindle-cell sarcoma. "Microscopic examination of various portions of the growth" (on the outer aspect of the middle of the left leg) showed spindle cells arranged "in bundles that interlaced each other in every direction; a layer of enlarged capillary blood vessels covered the surface of the growth, from which blood seemed to have been abundantly effused into the surrounding tissues, part being there decomposed, besides what escaped in the hemorrhages, thus giving rise to the black color," which was noticed in the growth. "Regarding that class of tumors called sarcomata, it has been observed that they tend to reappear after operation in the cicatrix or its neighborhood, rather than to spring up continuously in the same spot as do cancers proper; also, that they do not involve the lymphatic glands as certainly and promptly as cancers do. Spindle-celled sarcomata are found especially often in muscles, fasciæ and cutis. They are locally very infectious."—(*St. Louis Medical and Surgical Journal* for Jan. 1874.)

2. In the Philadelphia Hospital (service of Dr. H. C. Wood), a case of "Bright's Disease," with leucocythæmia and disease of the bone marrow occurred, with the following interesting post mortem history: "Spleen slightly enlarged; no lymphoid points visible in it; weight, nine and one-half ounces. Pancreas abnormally hard; weight, four and one-half ounces. Glands of axillæ and inguinal region somewhat hypertrophied, but scarcely larger than filberts; lymphatic structure elsewhere apparently not affected. Kidneys very small, offering on microscopical examination the usual lesions of contracted kidney. The tibia, when sawn open, offering apparently healthy marrow, approximating lemon-yellow, and normal in appearance. The marrow of the femora dark red, presenting the general appearances seen in cases of leucocythæmia elsewhere." Reported by Dr. H. C. Wood. The marrow of the femora, on microscopic examination, "showed an enormous increase of the so-called lymphoid elements, which have been described by Neumann as intermediate forms between the white and red blood-corpuscles. These lymphoid elements are quite small in size. Large cells with a distinct cell-wall were also seen, containing from eight to twenty blood-corpuscles. The fully formed red disks, although quite numerous, were less abundant than the intermediate lymphoid forms."—(*Medical Times*, Jan. 3, 1874.)

The point of interest in connection with this case is the absence of marked enlargement of the spleen or lymphatic glands; while both spleen and lymphatic glands were slightly above the natural size, the enlargement was quite insufficient to account for the leucocythæmia.

3. The following description of a case of gout is taken from the *Medical Record* of Jan. 1: "The case was interesting in the fact that it illustrated a wonderful development of the disease in a boy aged twenty, without any real cause, unless it was an hereditary tendency. His fingers had the appearance of a bunch of carrots; nearly all the joints of the body contained gouty deposits, and the helix of his ears was filled with tophi."

4. Mr. Bat. Smith, medical student, discusses the "Origin and Action of Miasm" in the *New Orleans Medical and Surgical Journal*

for Jan., 1874, and after rejecting the germ or parasite theory, says: "In view of the unsatisfactory results of the investigations of this parasite theory, at least as far as malaria is concerned, I am led to believe with Schwalbe, that the true cause of miasmatic diseases should be ascribed to some peculiar chemical substance of a gaseous nature, rather than to a low living organism." Basing his conclusions upon the results of numerous investigations made by himself in the malarial districts of Central America, he pronounces miasm to be a gas generated by the decomposition of certain vegetable substances. The sun's rays, and the molecular interchange in the vegetable matter, from large quantities of oxygen, destroy or neutralize this gas, either wholly or in part. After sunset the gas attains a sufficient degree of concentration to act detrimentally upon the human system. The action of the poison is most intense during the night. It is suspended near the surface of the earth.

5. Dr. Eberth, of Zurich, states: "That he has found in ordinary sweat, small, oval-shaped bacteria, which are frequently united in strings of two or three, and endowed with rather active movements. In spots covered with hair they attach to the hair, and often form thick layers, whilst others penetrate into the hair, which then splits and breaks. Coloring by hæmatoxylon brings out the isolated bacteria, as well as those collected in the hair."

6. "Sir W. Jenner gives the details of the case of a man whose abdomen gave way at the umbilicus with a loud report, in a clinical lecture, delivered at the University College Hospital. In this case the gas was in the peritoneal cavity, and he had previously been tapped. The wound did not heal, and when the gas rapidly accumulated the weak spot ruptured. The real causes of the man's troubles were an impediment to the flow of blood through the portal vein, and an impediment to the escape of bile from the hepatic duct. * * * The patient had had hæmatemesis, and suffered from perforation of the bowel, which led to peritonitis."

7. Dr. Samuel B. Ward relates two cases of arterial embolism occurring in the extremities, which are worth summarizing:

Case I. An Irishman, aged thirty-two, married, plasterer by trade; has never had rheumatism, syphilis, or any serious (acute)

illness, except intermittent fever about fifteen years ago, and diphtheria eight years ago. August 6, 1873, after "drinking freely," the patient was taken with gastric distention, followed by endocarditis. August 11, embolism of the "right popliteal artery by a clot or vegetation from the valves of the heart," suddenly made its appearance, as attested by the fact that the limb became "white as marble, and apparently as bloodless; and the surface, from a hand's breadth above the knee to the extremity of the toes, perfectly cold. Pulsation could be felt in both tibials of the left limb; on the right side no pulsation could be felt below the opening in the adductor magnus for the passage of the femoral." On the 13th the symptoms were much improved, and, says Dr. Ward, "there is every appearance that collateral circulation is being established." On the 14th, "condition of limb slightly worse; no pulsation can be felt in the femoral below the middle of the thigh." On the 15th, "no pulsation can be felt in the femoral, even at the groin," and it was evident that a clot filled the artery above Poupart's ligament. The signs of gangrene appeared promptly. On the 22d trismus manifested itself, and on the 23d the patient died.

No post-mortem allowed.

Case 2. "Mrs. M., American, aged 31, married, having always enjoyed fair health, went through her first and second pregnancies and confinements without any unusual occurrence. Has never had any heart disease. Twenty-four hours after her second labor, which occurred January 10, 1865, she had a severe chill, and two or three hours later was suddenly seized with intense pain in the right leg, which soon amounted to perfect agony. On examination the foot and leg were found to be very pale up to a line about three inches below the knee, and through the same extent coldness, with a very great diminution, if not total absence, of sensation, was noticed." * * * "There was a single purplish streak from the knee down the outer side of the leg on to the foot." Gangrene promptly made its appearance, and "the integument sloughed off, until the muscles, tendons, ligaments and bone were all exposed" from the knee to the foot, with the exception of the tissues corresponding to the "purplish streak" already mentioned. One month after the accident, the slough had separated, and granulations appeared; at the end of three months she was able to leave her bed; at the end of five months began to go out of doors, and at present a large scar is to be seen, and a granulating, rather smaller than a silver dollar, which has never healed.

8. Dr. Lente, of Cold Spring, N. Y., reports a case of embolism of the pulmonary artery, with the following history: "In Jan. 1, 1874, a woman was thrown out of a sleigh, and received a fracture of the tibia, which penetrated the knee joint, and at the same time received a severe injury in the chest from the seat of the sleigh." Three weeks after the injury the doctor "was sent for urgently, but, in his absence, his assistant visited the patient. She was found blanched, pulseless, and complaining of nausea, and headache, and pains in different parts of the body. The patient was attacked about one o'clock, and died about seven."

"Autopsy thirty hours after death. The lungs were found engorged with blood, with the exception of a small place anteriorly. The pulmonary artery on the right side was completely plugged up by an embolus. On the left side the artery was nearly in the same condition, but it allowed a small amount of blood to pass. From this obstruction no blood found its way into the left side of the heart, and, in this way, all the symptoms of internal hæmorrhage were produced. There was no appreciable dyspnœa. There was no injury of the sternum, and no lesion of the heart to account for the embolus."

